# Unrecognized myocardial infarction by echocardiography in relation to infarct characteristics as assessed by cardiovascular magnetic resonance imaging

**DOI:** 10.1186/1532-429X-14-S1-O36

**Published:** 2012-02-01

**Authors:** Caroline Jaarsma, Simon Schalla, Emile C Cheriex, Martijn Smulders, Ivo M van Dongen, Patricia J Nelemans, Joachim E Wildberger, Harry J Crijns, Sebastiaan C Bekkers

**Affiliations:** 1 Cardiology, Maastricht University Medical Center, Maastricht, Netherlands; 2 Radiology, Maastricht University Medical Center, Maastricht, Netherlands; 3 Cardiovascular Research Institute Maastricht, Maastricht University, Maastricht, Netherlands; 4 Epidemiology, Maastricht University, Maastricht, Netherlands

## Background

Accurate recognition of myocardial infarction (MI) is important, because of the benefits of immediate and correct treatment. Despite being the method of choice, echocardiographic assessment of segmental wall motion abnormalities (SWMA) in MI can be difficult and depends on operator skills, experience, and image quality. The purpose of this study was to investigate the echocardiographic detection of SWMA by experienced echocardiographers in relation to underlying infarct characteristics as assessed with delayed-enhancement cardiovascular magnetic resonance imaging (DE-CMR).

## Methods

In 88 consecutively and prospectively enrolled first ST-segment elevation MI patients, 141 echocardiograms, performed 2 (interquartile range [IQR] 1-4) days (acute, n=61) and 102 (IQR 92-112) days post-MI (chronic, n=80), were available for evaluation. Pooled with echocardiograms of 36 healthy controls (absence of coronary artery disease and normal CMR), two experienced echocardiographers, who were blinded to patient and DE-CMR data, randomly evaluated all 177 echocardiograms for SWMA. This was compared with infarct characteristics, as assessed with DE-CMR performed 104±11 days post-MI.

## Results

**Figure 1:**
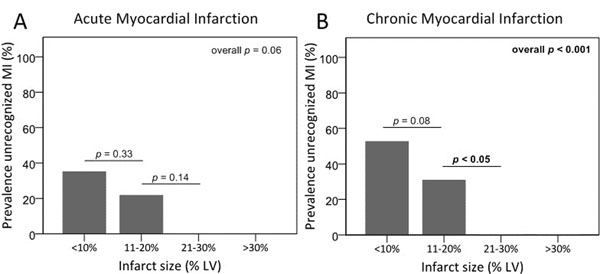
Unrecognized myocardial infarction in relation to infarct size.

**Figure 2:**
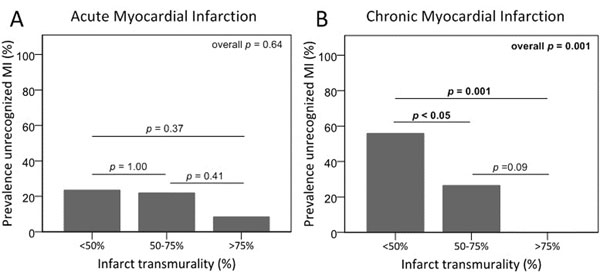
Unrecognized myocardial infarction in relation to infarct transmurality.

In the MI group, mean age was 59±11 years and 74% were men vs. 43±12 years and 56% men in healthy controls. The infarct-related artery (IRA) was the LAD, LCx and RCA in 31%, 12%, and 57% of patients, respectively. All patients showed hyperenhancement on DE-CMR matching the IRA territory. The median infarct size was 11% (IQR 5-19) with a mean infarct transmurality of 57±16%. The sensitivity of echocardiography to detect SWMA was 80.3% in the acute, and 65.0% in the chronic phase; the specificity was 80.6%. In patients with unrecognized MI, infarcts were smaller (6% [IQR 3-12] vs. 15% [IQR 9-23], p<0.001, Figure 1) and less transmural (50±14% vs. 61±15%, p<0.001, Figure 2), left ventricular ejection fraction (LVEF) was higher (59±5% vs. 46±8%, p<0.001), and MI localization was more often non-anterior than anterior (83% vs. 63%, p<0.05). The strongest determinants of echocardiographic recognition of acute MI were LVEF (odds ratio per unit increase [OR] 0.77 (95% confidence interval [CI] 0.64-0.92), p<0.01), and LVEF and infarct size of chronic MI (OR 0.63 [95%CI 0.49-0.81], p<0.001 and OR 1.20 [95%CI 1.00-1.45], p=0.06, respectively). Segmental analysis showed a significant increase in SWMA with increasing infarct transmurality, both in acute as well as chronic MI (p<0.001).

## Conclusions

The sensitivity of echocardiography to detect MI was higher in acute than chronic infarctions. Unrecognized MI were smaller, less transmural, more often located non-anterior and related to a higher LVEF.

## Funding

None.

